# Moderate Exercise Combined with Enriched Environment Enhances Learning and Memory through BDNF/TrkB Signaling Pathway in Rats

**DOI:** 10.3390/ijerph18168283

**Published:** 2021-08-05

**Authors:** Liyuan Xu, Linna Zhu, Lina Zhu, Dandan Chen, Kelong Cai, Zhimei Liu, Aiguo Chen

**Affiliations:** College of Physical Education, Yangzhou University, Yangzhou 225127, China; MX120180363@yzu.edu.cn (L.X.); MX120180361@yzu.edu.cn (L.Z.); zhulina827@mail.bnu.edu.cn (L.Z.); DX120200078@yzu.edu.cn (D.C.); MX120170353@yzu.edu.cn (K.C.); DX120200076@yzu.edu.cn (Z.L.)

**Keywords:** combined intervention, exercise, enriched environment, learning and memory ability, BDNF/TrkB signaling pathway

## Abstract

This study aimed to investigate the effects and potential mechanisms of exercise combined with an enriched environment on learning and memory in rats. Forty healthy male Wistar rats (7 weeks old) were randomly assigned into 4 groups (*N* = 10 in each group): control (C) group, treadmill exercise (TE) group, enriched environment (EE) group and the TE + EE group. The Morris water maze (MWM) test was used to evaluate the learning and memory ability in all rats after eight weeks of exposure in the different conditions. Moreover, we employed enzyme-linked immunosorbent assay (ELISA) to determine the expression of brain-derived neurotrophic factor (BDNF) and receptor tyrosine kinase B (TrkB) in the rats. The data showed that the escape latency and the number of platform crossings were significantly better in the TE + EE group compared to the TE, EE or C groups (*p* < 0.05). In addition, there was upregulation of BDNF and TrkB in rats in the TE + EE group compared to those in the TE, EE or C groups (*p* < 0.05). Taken together, the data robustly demonstrate that the combination of TE + EE enhances learning and memory ability and upregulates the expression of both BDNF and TrkB in rats. Thus, the BDNF/TrkB signaling pathway might be modulating the effect of exercise and enriched environment in improving learning and memory ability in rats.

## 1. Introduction

Learning and memory are fundamental features in the development and survival of humans and animals. Besides being critical for higher-order brain functions, they are intimately associated with behavioral and psychological consequences. Previous studies have associated the brain-derived neurotrophic factor (BDNF) signaling pathway with learning and memory [1]. BDNF exerts widespread effects throughout the central nervous system, thus mediating critical processes in learning and memory [2]. Furthermore, other reports have demonstrated a correlation between central and peripheral BDNF in rats and other animals as well as its ability to cross the blood–brain barrier [3,4,5]. Therefore, the peripheral BDNF might be a biomarker for learning and memory functions. BDNF binds to its specific receptor tyrosine kinase B (TrkB) to promote learning and memory performance and participates in the growth, differentiation and repair of neurons [6]. For instance, exogenous introduction of BDNF and its receptor TrkB agonist was shown to prevent stress-induced spatial memory deficits [7,8]. Understanding the role of the BDNF/TrkB signaling pathway in mediating learning and memory development remains of immense research interest. An increasing body of evidence from in vivo experiments has shown that enriched environment, physical exercise, learning experiences or social interactions induce changes in the BDNF/TrkB signaling pathway, resulting in learning and memory shifts.

The effects of physical exercise on memory retention and learning have been demonstrated both in animal models and humans [9,10,11]. Other studies have demonstrated the neuroprotective effect of regular physical exercise in improving learning and memory in healthy individuals [12,13]. Moreover, recent studies have associated exercise with an increased release of BDNF and TrkB, which are delivered to the brain and might play a role in learning and memory [14,15]. On the other hand, whereas regular physical activity benefits learning and memory over the whole life course, the benefits are dependent on aspects of physical exercise plans such as speed, time or slope [16,17,18]. For instance, moderate treadmill exercise was shown to positively modulate the concentrations of BDNF and its TrkB receptor in experimental animals [19,20]. Therefore, the different exercise intensities have different effects on learning and memory as well as BDNF/TrkB signaling activities. Thus, exercise dosage appears to be an important factor in the achievement of enhanced cognitive capabilities.

On the other hand, an enriched environment entails a combination of complex inanimate and social stimulations [21]. An enriched environment consists of physical exercise, novel stimulants and social interactions [22]. Previous findings showed that learning and memory impairment can be attenuated by a relatively short (a few weeks) exposure to environmental conditions; provision of sensory, exercise or cognitive stimulations; as well as sustained social interactions in rodents [23]. In addition, BDNF and TrkB have been shown to be upregulated in the brain in animals maintained in an enriched environment compared to those in impoverished conditions (isolation, no stimulation for physical or learning experiences) [24]. Moreover, an enriched environment could improve learning and memory, and the up-regulation of BDNF/TrkB is considered to be an important pathway in the improved features. One clear difference between treadmill exercise and voluntary exercise in the enriched environment is the ability to quantify the exercise plans for animals undergoing treadmill exercise. On the contrary, the amount of voluntary exercise in the enriched environment cannot be controlled [25].

Previous studies have demonstrated that either the enriched environment or exercise could be promising strategies in enhancing learning and memory as well as upregulation of the BDNF/TrkB signaling pathway, thus highlighting their therapeutic potential [26,27,28]. It has been shown that, compared to a single intervention, combined exercise and enriched environment interventions yield better effects in healthy animals [29,30,31,32]. However, data on the effect of forced exercise combined with voluntary exercise in an enriched environment remains scant. Besides, previous research on learning and memory and the BDNF/TrkB signaling pathway has focused on the effectiveness of a single intervention. Here, we evaluated the effect of a combination of exercise and enriched environment interventions on learning and memory ability, as well profiling the expression of the BDNF/TrkB signaling pathway in healthy Wistar rats. Based on the above research, we put forward the following hypothesis that the combination of treadmill exercise and enriched environment enhances the learning and memory ability by upregulating the concentrations of BDNF/TrkB in rats.

## 2. Experimental Procedures

### 2.1. Animals

Male Wistar rats (6 weeks old), purchased from Laboratory Animal Center (Yangzhou University) [certificate: SCXK (SU) 2017-0007], were used in this study. The study was approved by the Institutional Animal Care and Use Committee and Ethical Committee of Yangzhou University. Initially, the rats were housed in standard cages 46 × 30 × 16 cm (L × W × H), 5 per cage for 7 days. On day 8, 40 healthy rats were pulled together and then placed in the same environment, to allow random grouping. Then, all rats were randomly selected and assigned by an independent technician as follows: each rat was randomly picked up from the cage and assigned to the control (C) group, the treadmill exercise (TE) group, the enriched environment (EE) group and the treadmill exercise combined with enriched environment (TE + EE) group in sequence, and the assignment was repeated until all groups reached the designated number of animals (*N* = 10 in each group). In order to reduce the treadmill exercise stress, the rats in TE and TE + EE groups were allowed to adapt to treadmill exercise for one week period prior to the commencement of the experiments. The experimental protocol consisted of 40 min of daily exercise for 6 days and rest for 1 day. The rats were housed in groups in a controlled room (temperature 23 ± 2 °C; 12 h light/12 h dark cycle, light on at 6 PM and off at 6 AM). Except for the C and the TE groups, food and water were delivered from both sides of the cage. Sawdust bedding (SPF) was provided at approximately 2 cm depth. The rats were 8 weeks of age at the onset of the experiments.

### 2.2. Groups

#### 2.2.1. Control Group

**Figure 1:**
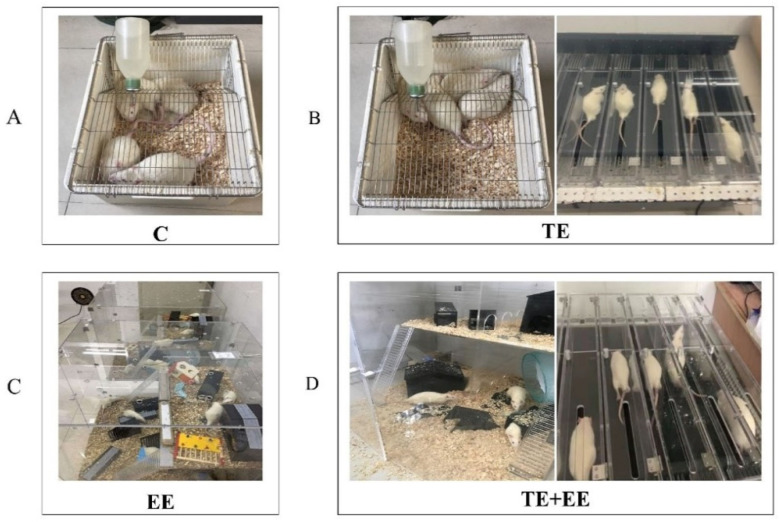
Study groups: (**A**) Control (C); (**B**) Treadmill Exercise (TE); (**C**) Enriched Environment (EE); (**D**) Treadmill Exercise Combined with Enriched Environment (TE + EE).

Five animals per cage were housed in standard cages as a control group. The cages had bedding, regular rate chow and plain boiled water (Figure 1).

#### 2.2.2. Treadmill Exercise

The rats were familiarized with the treadmill to eliminate any exercise-related stress. The rats were adapted to a running treadmill for 40 min daily for 6 days (running at a speed of 5 m/min for the 1st and 2nd days, 10 m/min for the 3rd and 4th days, and 20 m/min for the 5th and 6th days, with 0° inclination.). 

As indicated, exercise-induced benefits are dependent on various quantifiable plans of physical exercise such speed, time and slope. We performed regular moderate exercise with newfangled dynamics and exercise load standards as previously described (Bedford et al.).

Eight-week-old rats were then forced to run on a treadmill, with the 0° inclination, at a speed of 20 m/min for 40 min daily, 6 days a week, for 8 consecutive weeks [33].

#### 2.2.3. Enriched Environment

The rats in the EE group were housed for a whole day and then divided into 2 cages (83 × 83 × 83 cm) in order to promote social interaction. Each cage had two floors connected by ramps to promote physical exercise and movement. Various elements of different shapes and textures such as balls, stairs, cubes, tunnels, swings and wheels were placed in the cages and were available to the animals for the 8 weeks of the experiment. The objects in each cage were rotated once a week to stimulate sensory, motor and cognitive functions.

#### 2.2.4. Treadmill Exercise Combined with Enriched Environment

Like in the TE or EE group, one dimension of the intervention contained the treadmill while the other contained toys and food treats. Prior to the intervention, the rats underwent the same adaptive exercise as described in the TE group for 1 week. After 40 min on the treadmill exercise, the rats were kept in the enriched environment for the rest of the day.

### 2.3. Experimental Design

The rats in each group underwent the experimental protocols for 8 weeks (*N* = 10 per group). Thereafter, the animals were allowed to acclimatize to the laboratory environment for one day. 6-day Morris water maze (MWM) tests were employed to assess the learning and memory functions in the rats. Testing took place during the light phase of the light/dark cycle and the animals were immediately returned to cages after the tests. To prevent the 6-day MWM test from affecting the intervention effects, all rats were given the intervention for four extra days [34]. On the fifth day, the rats were anaesthetized with urethane and then blood was collected from the abdominal aorta and snap frozen (−80 °C) for further biochemical analysis.

### 2.4. Morris Water Maze

The Morris water maze (MWM) consisted of a circular galvanized steel pool (diameter = 120 cm; wall height = 50 cm), filled with water at 23 ± 1 °C. A small round escape platform (12 cm diameter) was fixed at the center of one quadrant, 2 cm beneath the water surface. The learning phase consisted of five training days, which randomly started at four different positions. We conducted four trials daily. The rats were placed into the pool facing the maze wall at fixed entry points. In case a rat could not find the platform within 120 s, the experimenter guided the rat to the platform [35]. The rat was then allowed to stay on the platform for 10s to memorize the location [36]. The water maze was surrounded by fixed clues. Moreover, the experimental room was kept invariable during the MWM testing [37]. We recorded the duration the rats spent searching for the platform in each quadrant, with an average of escape latency in the four quadrants as the final escape latency. On the sixth day, the platform was removed, and rats were placed in water to swim with a limitation of 60 s. We then recorded the number of times the rats crossed the exact place containing the submerged platform in each quadrant, with the average number of platform crossings in the four quadrants considered the probe trial of the day. Images of swimming rats were captured by a video camera placed above the center of the pool, which was connected to a computer system running specialized tracking software (ANY-maze, Stoelting Co., Wood Dale, IL, USA).

### 2.5. ELISA for Plasma BDNF and TrkB

Using the enzyme-linked immunosorbent assay (ELISA), we quantified the concentrations of BDNF and TrkB in the plasma of the rats, following the manufacturer’s instructions (Shanghai Enzyme-linked Biotechnology Co., Ltd, Shanghai, China). Dispensed antigen standards and samples were added to each well in the 96-well plates, precoated with primary antibodies. After the addition of biotin and enzyme conjugate reagents into the wells, the plates were incubated at 37 °C for 60 min. We then washed the plates five times in distilled water. Within 15 min of chromogenic reaction, the absorbance was read at 450 nm using a microplate reader (Nano Drop ND-1000, Waltham, MA, USA).

### 2.6. Statistical Analysis

Data were analyzed using JAMOVI (version 1.6.1) statistical software. A *p* < 0.05 was considered statistically significant. For the MWM test acquisition, the average escape latency time(s) to reach the platform per day was analyzed by repeated measure analysis with days as the within-subjects factor and treatment (C, TE, EE, TE + EE) as the between-subjects factor. The Tukey post-hoc tests were used to evaluate pair-wise differences between the group means. For the probe trial, the number of platform crossings was analyzed using a two-way ANOVA with exercise factors (running versus not running) and enrichment (enriched versus not enriched); data were not corrected. In addition, we used the post-hoc tests to evaluate pair-wise differences between the group means. The BDNF and TrkB concentrations were analyzed using a two-way ANOVA using exercise factors (exercise versus not exercise) and enriched environment (enriched versus non enriched environment); data were not corrected. Furthermore, we used post-hoc tests to evaluate pair-wise differences between the group means.

## 3. Results

### 3.1. Behavioral Performance: The Morris Water Maze (MWM)

**Figure 2:**
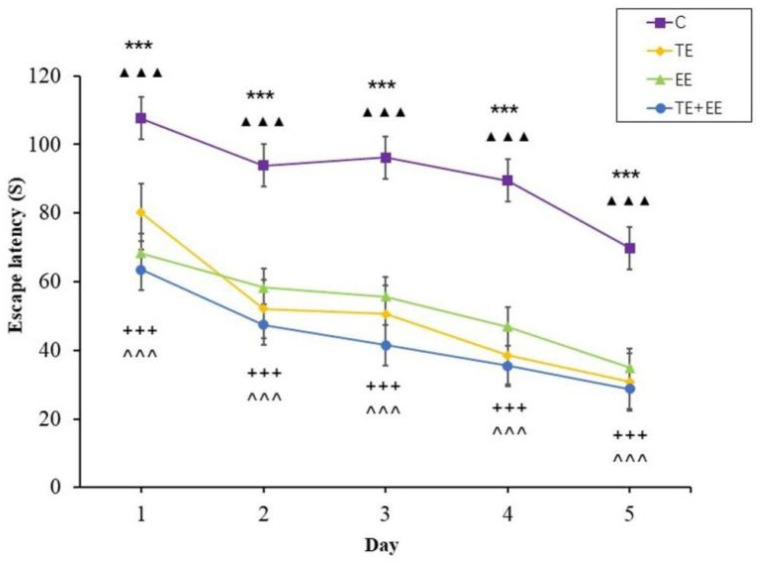
Average escape latency in the Control (C), Treadmill Exercise (TE), Enriched Environment (EE) and Treadmill Exercise Combined with Enriched Environment (TE + EE) groups (*N* = 10, $\bar{x}\pm s$). Note: EE group versus C group, *** *p* < 0.001; TE group versus C group, ▲▲▲ *p* < 0.001; TE + EE group versus C group, + + + *p* < 0.001; TE + EE group versus EE group, ^ ^ ^ *p* < 0.001.

The escape latency data showed that the time effect [*F* (4,144) = 68.77, *p* < 0.001, partial *η^2^* = 0.66] and the group effect [*F* (3,36) = 103, *p* < 0.001, partial *η^2^* = 0.90] were statistically significant, but the time × group interaction effect [*F* (12,144) = 1.69, *p* > 0.05, partial *η^2^* = 0.12] was not statistically significant. In addition, the escape latency of each group decreased with the increase in days. Further analysis showed that the TE, EE and TE + EE groups’ escape latency were better compared to that of the C group (*p* < 0.001) while that of the TE + EE group was better than that of the EE or C groups (*p* < 0.001) (Figure 2). 

**Figure 3:**
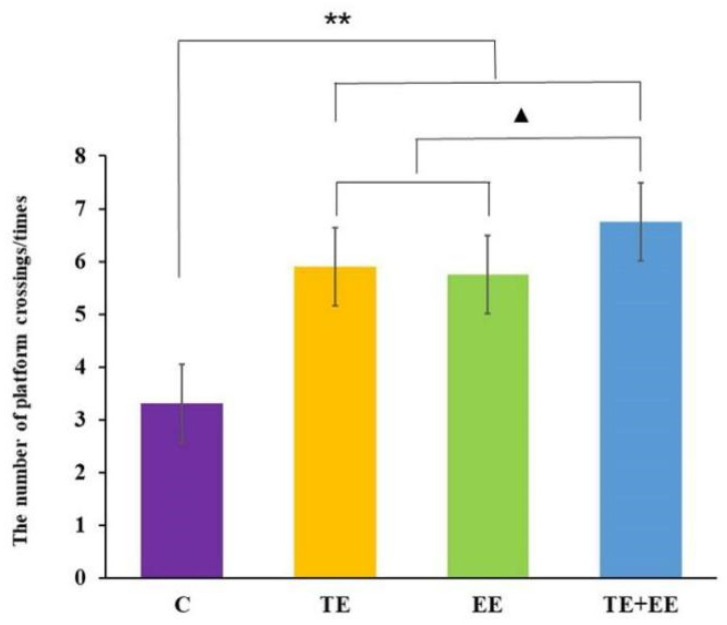
Average number of platform crossings in the Control (C), Treadmill Exercise (TE), Enriched Environment (EE) and Treadmill Exercise Combined with Enriched Environment (TE + EE) groups (*N* = 10, $\bar{x}\pm s$). Note: compared with the C group, ** *p* < 0.01; compared with TE + EE group, ^▲^ *p* < 0.05.

Data from the number of platform crossings showed a statistically significant difference between the exercise effect [*F* (1,36) = 56.90, *p* < 0.001, partial *η^2^* = 0.61], the enriched environment effect [*F* (1,36) = 47.80, *p* < 0.001, partial *η^2^* = 0.57] and the interaction effect of the exercise × enriched environment [*F* (1,36) = 11.20, *p* < 0.01, partial *η^2^* = 0.24] in the probe trail of the rats. Further post-hoc analysis showed that the rats in the TE + EE, TE, or EE groups had a significantly increased number of platform crossings compared with those in the C group (*p* < 0.001). Unlike between the TE and EE groups, the number of platform crossings of the rats in the TE + EE group was significantly higher than those in the TE or EE groups (*p* < 0.05) (Figure 3).

### 3.2. BDNF and TrkB in Plasma

To define the mechanism of learning and memory in rats exposed to the combination of exercise and an enriched environment, we interrogated the expression profile of the BDNF and TrkB in the rats’ plasma.

Our data demonstrated that there was a significant difference between the exercise effect [*F* (1,36) = 28.95, *p* < 0.01, partial *η^2^* = 0.45] and the enriched environment effect [*F* (1,36) = 27.02, *p* < 0.01, partial *η^2^* = 0.43] as well as the interaction effect [*F* (1,36) = 5.03, *p* < 0.05, partial *η^2^* = 0.12] in the expression of BDNF in the rats. Post-hoc analysis showed that the BDNF concentration was significantly increased in the TE + EE, TE or EE groups compared with those in the C group (*p* < 0.01). In addition, the BDNF concentration in the TE + EE group was significantly higher than that in the TE or EE groups (*p* < 0.05). On the contrary, there was no statistically significant difference in the BDNF concentration between the TE and EE groups (*p* > 0.05).

Similarly, the data showed a significant difference between the exercise effect [*F* (1,36) = 53.44, *p* < 0.01, partial *η^2^* = 0.60] and the enriched environment effect [*F* (1,36) = 27.88, *p* < 0.01, partial *η^2^* = 0.44] as well as the interaction effect [*F* (1,36) = 4.99, *p* < 0.05, partial *η^2^* = 0.12] in the expression of TrkB in the rats. Post-hoc analysis showed that the TrkB concentration was significantly upregulated in the TE + EE, TE or EE groups compared with those in the C group (*p* < 0.01). Moreover, the TrkB concentration in the TE + EE group was significantly higher than that in the TE or EE groups (*p* < 0.05). There was, however, no statistically significant difference in the TrkB concentration between the TE and EE groups (*p* > 0.05) (Table 1).

## 4. Discussion

This study investigated the effects and mechanisms of moderate exercise combined with an enriched environment on learning and memory in rats. We further interrogated the role of the BDNF/TrkB signaling pathway in mediating learning and memory effects in rats. Our data demonstrate that exercise, enriched environment or exercise combined with an enriched environment intervention improves the learning and memory ability in rats and the effect is mediated by BDNF/TrkB. Interestingly, exercise combined with the enriched environment conferred the best effect.

Our study showed that exercise could improve learning and memory ability and increase the expression of BDNF and TrkB in rats. Our findings are consistent with previous data which used exercise intervention methods alone in rats. Many studies have shown that moderate treadmill exercise has a positive effect on learning and memory. However, the intensity of the exercise determines the optimal learning and memory effects [38,39]. Control of the frequency, duration and intensity of exercise, which are essential aspects in evaluating the beneficial effects of exercise, is more feasible with treadmill exercise, while moderate-intensity running (speed up to 21 m/min) positively affects information acquisition, learning and memory [40,41]. In our study, we used treadmill workouts with defined parameters (such as intensity, duration and cycle). This would explain the fact that the escape latency of the rats in the TE group was lower than that of the C group. In addition, recent data have demonstrated a positive correlation between BDNF expression and different types of physical exercises [42,43,44,45,46]. Similarly, exercise-induced elevation of brain BDNF was reported to be intensity-dependent [47,48]. Moreover, the upregulation of BDNF expression might also be dependent on the duration and frequency of exercise [49,50,51]. This phenomenon has been shown to not only be beneficial to the peripheral nervous system, but also to the central nervous system [52].

We demonstrated that the rats in the EE group exhibited significantly improved learning and memory as well as upregulation in the BDNF/TrkB pathway. Novel stimulants, social interactions and physical exercise are components of the enriched environment. Our study used novel objects, and their rearrangement triggered fresh exploration of the enriched environment by the EE and TE + EE rats. Furthermore, compared with the standard squirrel cage, the enriched environment box had more companions and doorways for communication. Thus, it is possible that the effects of an enriched environment are a function of interaction with the cage mates [53]. In addition, physical exercise has been proposed as a critical component of an enriched environment. However, there is a difference between voluntary exercise in an enriched environment and treadmill exercise. Unlike in a previous study, our findings showed that there was no difference in the learning and memory of rats in the TE and EE groups [54]. This was probably because there was no autonomous runner in the enriched environment. In our study, the two layers of rats in the enriched environment were connected by ramps and contained autonomous runners, which obviously promoted their exercise. In addition, the enriched environment has been shown to increase brain and blood BDNF concentration [55,56,57]. Previous studies reported that BDNF signaling is closely associated with learning and memory functions. The acquisition of learning and memory is accompanied by an increase in the BDNF gene expression in specific brain regions. Blocking the effect of BDNF would lead to declined learning and memory abilities. An appealing feature in the use of BDNF as an indicator for effective enrichment is the correlation between the blood and brain BDNF concentrations, as BDNF can cross the blood–brain barrier [58,59]. Hence, blood-based measures of BDNF have been used as a proxy for brain BDNF [60], allowing for a less invasive assessment of brain changes. BDNF through TrkB receptors contributes to the proliferation, survival and differentiation of neurons in the hippocampus and other brain regions closely related to learning and memory, as well as promoting the induction of long-term potentiation and improving the ability of learning and memory in experimental animals [61]. Long-term potentiation in the hippocampus is an activity-dependent modification of synaptic strength and is considered a potential cellular mechanism underlying learning and memory. Our study demonstrated that the rats housed in total exercise and enriched environment conditions had changes in behavioral and physiological outcomes.

To improve the effectiveness of a single intervention method, the combination of two effective behavioral strategies, such as exercise combined with an enriched environment, presents a feasible alternative. Our analysis showed that exercise combined with an enriched environment intervention has a significant effect in improving the learning and memory ability as well as the expression of the BDNF/TrkB signaling pathway in rats. Compared with a single intervention method, exercise combined with a rich environment confers superior effects on improving the learning and memory of rats. In agreement, Wang Chaolei et al. showed that an enriched environment intervention was slightly better than a swimming intervention, while swimming combined with rich environmental intervention was slightly better than enriched environment or swimming interventions [62]. On the other hand, previous studies have assessed the molecular mechanisms involved in the regulation of the BDNF/TrkB signaling pathway. For instance, Du Mingyang et al. showed that memantine and enriched environment therapy could effectively improve the learning and memory abilities in rapidly aging mice. Moreover, it increases the expression of BDNF and TrkB in the hippocampus [63]. Nasroallah Moradi Kor showed that combined enrichment of spirulina or combined exercise of spirulina has a synergistic effect on the hippocampal BDNF levels and dendritic morphology [64]. 

A number of limitations need to be noted regarding the present study. Firstly, our study only included male rats and could not be duplicated in female rats. It is reported that estrogen might affect the behavior of female rats [65,66]. Secondly, we only included healthy rats. It is possible that these results might not be applicable to other groups with cognitive dysfunctional model rats. Future studies need to assess cognitive ability after intervention in impaired models to provide early treatment strategies. Lastly, although our data robustly demonstrates that 8-week treadmill exercise can improve the learning and memory ability and upregulated expression of BDNF/TrkB in rats, previous literature proposed that a longer intervention period may stabilize the intervention effect on learning and memory [67,68]. Future studies need to investigate the effects of longer intervention cycles on learning and memory, as well as related mechanisms. In short, these limitations mean that the study findings need to be interpreted cautiously. 

## 5. Conclusions

Taken together, our data robustly demonstrates that exercise combined with an enriched environment can improve the learning and memory ability in rats. The improve learning and memory ability might be mediated by the upregulated expression of BDNF/TrkB.

## Figures and Tables

**Table 1:** Comparison of the plasma concentrations of BDNF and TrkB in the groups ($\bar{x}\pm s$).

Group	*N*	BDNF	TrkB
C	10	492.22 ± 77.03	1538.34 ± 133.86
TE	10	707.19 ± 63.63 **^▲^	2049.94 ± 122.38 **^▲^
EE	10	702.05 ± 106.30 **^▲^	1941.09 ± 239.68 **^▲^
TE + EE	10	790.49 ± 102.57 **	2213.22 ± 156.81 **

Note: compared with the C group, ** *p* < 0.01; compared with TE + EE group, ^▲^ *p* < 0.05. Study groups: Control (C); Treadmill Exercise (TE); Enriched Environment (EE); Treadmill Exercise Combined with Enriched Environment (TE + EE).

## Data Availability

The raw data supporting the conclusions of this article will be made available by the authors, without undue reservation.
